# The unique structure of the highly conserved PPLP region in HIV-1 Vif is critical for the formation of APOBEC3 recognition interfaces

**DOI:** 10.1128/mbio.03332-24

**Published:** 2025-01-21

**Authors:** Yasumasa Iwatani, Kazuhiro Matsuoka, Hirotaka Ode, Mai Kubota, Yoshihiro Nakata, Yuka Setoyama, Kanako Kojima, Mayumi Imahashi, Yoshiyuki Yokomaku

**Affiliations:** 1 Department of Infectious Diseases and Immunology, Clinical Research Center, National Hospital Organization Nagoya Medical Center, Nagoya, Aichi, Japan; 2 Department of AIDS Research, Nagoya University Graduate School of Medicine, Nagoya, Aichi, Japan; University of Pittsburgh School of Medicine, Pittsburgh, Pennsylvania, USA; University of California, San Francisco, San Francisco, California, USA

**Keywords:** APOBEC3, HIV-1, Vif, PPLP, ubiquitination, antiviral factor, structure

## Abstract

The human cellular cytidine deaminases APOBEC3s (A3s) inhibit virion infectivity factor (Vif)-deficient HIV-1 replication. However, virus-encoded Vifs abolish this defense system by specifically recruiting A3s to an E3 ubiquitin ligase complex to induce their degradation. The highly conserved Vif PPLP motif is critical for the Vif-mediated antagonism of A3s and is believed to be important for Vif multimerization. However, how the PPLP motif dictates the functions of Vif remains unclear. Here, we aimed to elucidate this mechanism using biochemical and structural biology approaches. First, we found that no stable Vif multimer complexes formed in our tandem coimmunoprecipitation assays. Next, a series of Vif truncation mutants were constructed, and the short α-helix α6 just downstream of PPLP was found to be the smallest fragment essential for efficient A3G degradation in cells. *In silico* structural analysis suggested that PPLP-α6 adopts a stable L-shaped conformation when complexed in Vif/CBF-β and contributes to the structural integrity of Vif. *In vitro* ubiquitination assays with recombinant proteins confirmed that PPLP-α6 is necessary to form the functional complex of the E3 ligase adaptor of Vif/CBF-β/elongin B/elongin C. Additionally, mutations of the highly conserved PPLP-α6 hydrophobic residues severely disrupted Vif function. In the Vif structure, PPLP-α6 is positioned behind α1-α2 that constitutes the A3-binding Vif interfaces. Therefore, both the PPLP motif and α6 play critical allosteric roles in maintaining the integrity of the A3 interaction interfaces. Our findings will also provide important data for the design of novel anti-HIV-1 compounds that disrupt the A3-binding Vif interfaces.

**IMPORTANCE:**

The APOBEC3 (A3) family enzymes potently block the replication of retroviruses, such as HIV-1. However, HIV-1 expresses Vif, a small multifaceted protein that binds and specifically eliminates A3s in infected cells via ubiquitination–proteasome degradation. Thus, A3–Vif interactions are attractive targets for anti-HIV-1 drug development. The Vif PPLP motif that is distal from these interfaces is necessary for A3 degradation; however, the mechanism by which PPLP participates in A3 degradation is unknown. In this study, we performed biochemical and structural biology analyses to elucidate the underlying mechanisms involved. We found that the PPLP motif, in addition to the short downstream fragment α6, forms a stable L-shaped conformation and acts as a scaffold for the A3 recognition interfaces. Importantly, mutations in α6 abolished Vif function to antagonize multiple A3 family enzymes. These findings provide important data for the development of novel HIV-1 inhibitors that utilize A3s as cellular defense enzymes.

## INTRODUCTION

The human immunodeficiency virus type 1 (HIV-1) genome encodes virion infectivity factor (Vif), which antagonizes the antiretroviral effects of the human APOBEC3 (A3) family of proteins for its efficient viral replication in natural hosts (reviewed in references 1, 2). In HIV-1-infected cells, the Vif protein forms a thermodynamically stable heterodimer complex with core-binding factor subunit beta (CBF-β) (3–7) and recruits A3 proteins to a cullin5 (Cul5)-based E3 ubiquitin ligase complex with the elongin B (EloB) and elongin C (EloC) adaptor (hereafter referred to as EloB/C), which subsequently promotes A3 proteasomal degradation (8). The A3 family of cellular cytidine deaminases comprises seven members: A, B, C, D, F, G, and H. On the basis of their structure, the Vif-interaction A3 sites can be classified as one of three different types: A3C, A3D, and A3F (A3C/D/F); A3G; and A3H. These three distinct structural interfaces on A3 proteins are recognized by a single Vif molecule (reviewed in references 2, 9).

Vif is a small intrinsically disordered protein with a molecular weight of approximately 23 kDa and is composed of approximately 192–216 amino acids, depending on the HIV-1 subtype. The N-terminal half of Vif is involved mainly in the interaction with A3, whereas the C-terminal half contains three patched motifs that bind Cul5, EloC, and A3F (reviewed in 9). Three distinct Vif regions are responsible for A3 interactions: ^14^DRMR^17^, E76, W79, and ^171^EDRW^174^ for A3C/D/F (10–13); K22, S23, K26, and ^40^YRHHY^44^ for A3G (12–15); and Y30, G37, F39, ^40^YRHHY^44^, E45, E54 G60, A62, R63, R90, and R93 for A3H and its ortholog chimpanzee A3H (cA3H) (16–19). These regions are located predominantly in α-helix 1 (α1) of Vif. Interestingly, a recent computational analysis of Vif sequences revealed significant spatial and temporal variations in the mutational landscape of HIV-1 Vif (20), although residues for certain functional motifs are highly conserved, including the A3-binding motifs, the BC-box motif (^144^SLQYLA^149^) responsible for EloC binding (21), and the HCCH motif (^108^HX_5_CX_17-18_CX_3-5_H^139^), which coordinates Zn^2+^ for interaction with Cul5 (22).

**Fig 1:**
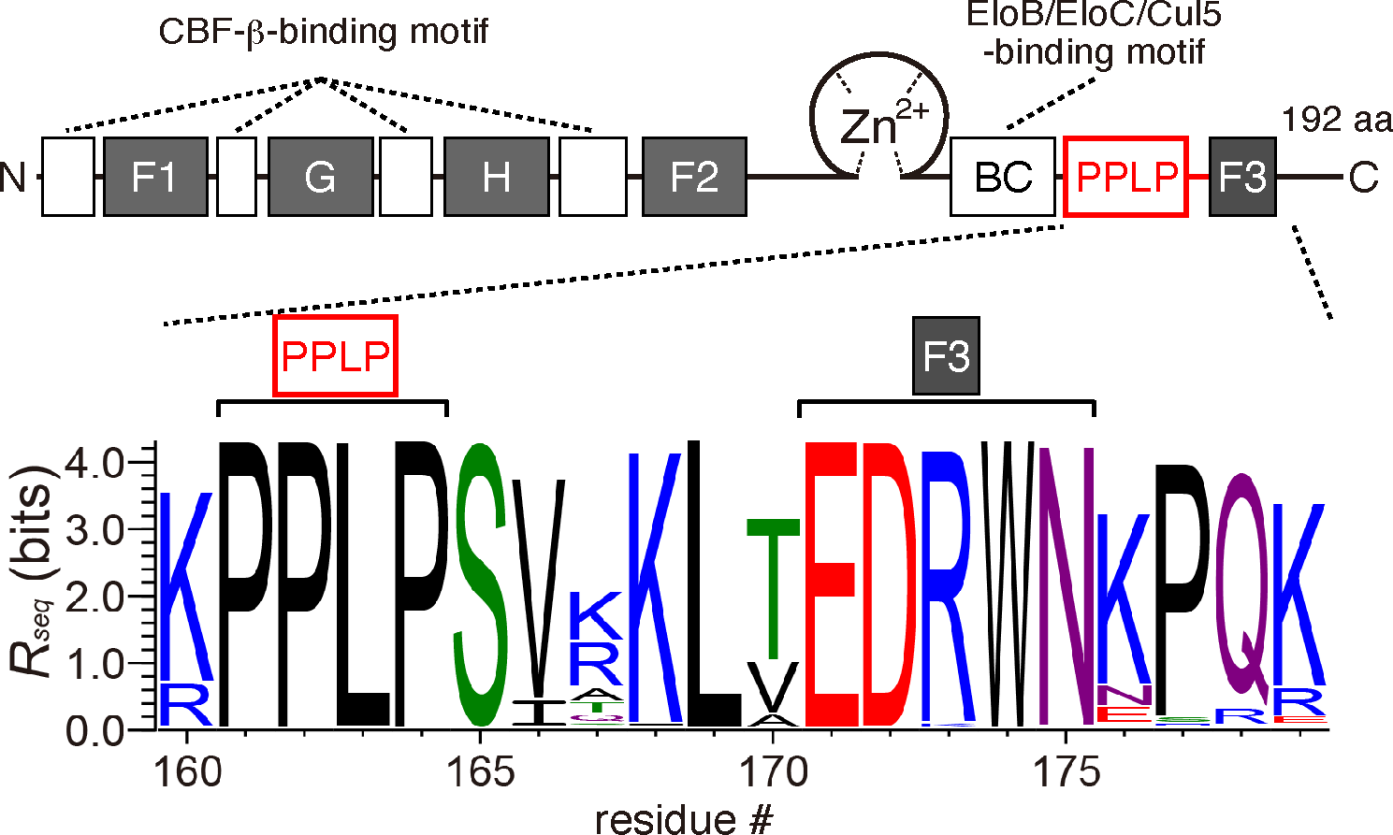
Conservation of the amino acid sequence in the HIV-1/SIVcpz Vif PPLP-F3 region. A diagram of the HIV-1 Vif functional domains is shown at the top. Three discontinuous motifs, F1, F2, and F3, and the G motif are important for interaction with A3F and A3G, respectively. The H box, Zn^2+^-coordination motif, and BC box are critical for interactions with A3H, Cul5, and EloC, respectively. The conservation rate at each position (residue #) around the PPLP-F3 region was analyzed using the WebLogo 3.4 program and is shown on the *y*-axis as *R_seq_* values (bits). A total of 8,013 HIV-1 sequences and 26 SIVcpz Vif sequences were aligned. Amino acids are colored according to their chemical properties: polar (green), neutral (purple), basic (blue), acidic (red), and hydrophobic (black).

In addition, a unique tetrapeptide motif, PPLP (residues 161–164), is highly conserved among the Vifs in HIV-1 (20), and the simian immunodeficiency virus was isolated from naturally infected chimpanzees (SIVcpz) (Fig. 1) (23). As this PPLP sequence is highly conserved, the importance of this unique proline-rich motif in Vif was identified in an earlier study (24). To date, several studies indicate that the HIV-1 PPLP motif is necessary for A3G degradation in HIV-1-infected cells (25, 26). Additionally, Vif proteins reportedly form stable homo-oligomers in both cell-free systems and cells, and mutations in the PPLP motif significantly impair the ability of Vif proteins to interact with each other (27–30). It was previously found that a 12-amino acid peptide containing the PPLP motif inhibited Vif and suppressed HIV-1 replication in MT-2 and H9 cells (28, 29), in which A3G is constitutively expressed (31). Hence, it is currently believed that the PPLP may play a critical role in Vif protein homo-oligomerization, although it remains unclear how Vif multimerization is linked to efficient Vif-mediated A3 degradation (28–30). However, three-dimensional structures of the A3 and Vif/CBF-β/EloB/C (VCBC) complexes were recently elucidated by cryo-electron microscopy (cryo-EM), in which no stable Vif dimers or direct interactions with the PPLP motif were observed (10, 14–16). These structural data prompted questions about whether the PPLP motif is intrinsically involved in Vif multimerization and what its possible role may be.

In this study, we first reassessed whether PPLP is involved in the multimerization of HIV-1 Vif in cells via tandem coimmunoprecipitation (co-IP) assays. Additionally, to explore the fundamental roles of PPLP, the minimal essential Vif sequence downstream of PPLP was determined by constructing a series of truncated Vif mutants. The results demonstrated that the short α-helix α6 (residues 165–171) just downstream of PPLP is the smallest fragment required for efficient A3G degradation. *In silico* structural analysis of the Vif/CBF-β complex was also performed to understand why PPLP-α6 is required for Vif function. Using recombinant proteins, we reconstituted A3 polyubiquitination systems *in vitro* and assessed the effects of PPLP-α6 mutations on VCBC complex formation and A3 ubiquitination. The results revealed that both the PPLP motif and α6 are required to form a functional VCBC complex for A3 ubiquitination, as these features may maintain the local conformational integrity of the Vif interface structures so that the three types of A3 family proteins are recognized.

## RESULTS

### Reassessment of PPLP-mediated Vif multimerization

**Fig 2:**
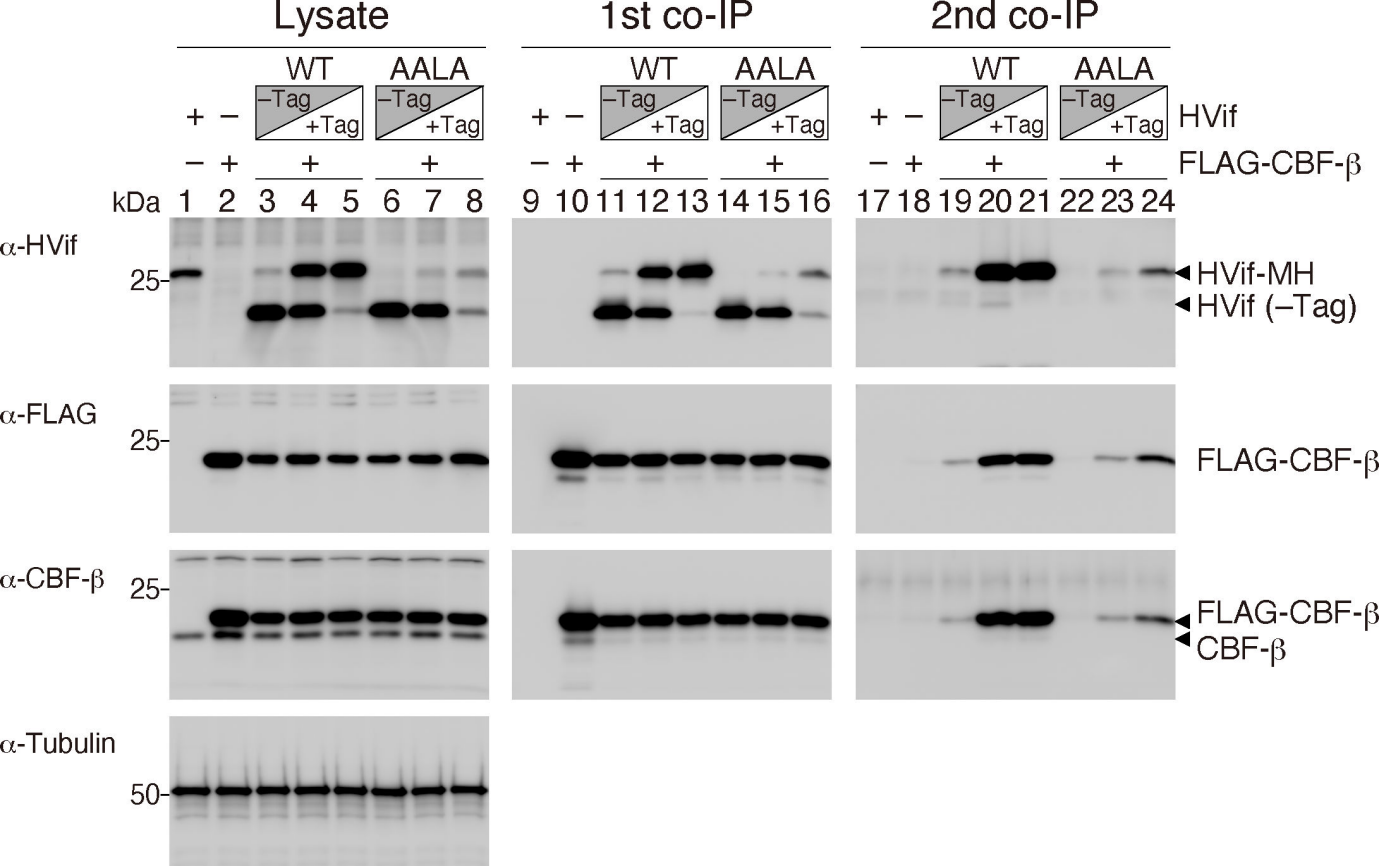
Analysis of the HIV-1 Vif homo-oligomers in cells by two-step co-IP. WT HVif (WT), or the HVif AALA mutant (AALA) carrying a C-terminal myc-HIS (MH) tag (+Tag) was coexpressed with untagged WT HVif (–Tag) in the presence or absence of FLAG-tagged CBF-β (FLAG-CBF-β) in 293T cells. The HVif (–Tag)/HVif-MH (+Tag) expression plasmids were used at the following ratios: 19:1 (lanes 3, 6, 11, 14, 19, and 22); 1:1 (lanes 4, 7, 12, 15, 20, and 23); and 1:19 (lanes 5, 8, 13, 16, 21, and 24). For the controls, only HVif-MH (+Tag) (lanes 1, 9, and 17) or only FLAG-CBF-β (lanes 2, 10, and 18) were expressed. The HVif-CBF-β complexes were coimmunoprecipitated with anti-FLAG M2 beads and eluted with a 3× FLAG peptide (first co-IP). The eluates were further pulled down using an anti-HIS rabbit polyclonal antibody (second co-IP). The total cell lysates (Lysate) and co-IP fractions (the first co-IP and the second co-IP) were analyzed by immunoblotting with anti-Vif (α-HVif), anti-FLAG (α-FLAG), or anti-CBF-β (α-CBF-β) monoclonal antibodies (mAbs). The anti-β tubulin polyclonal antibody (α-tubulin) was used as the loading control.

Initially, to reassess whether the PPLP motif plays a critical role in homomultimerization, we performed a two-step co-IP assay using human embryo kidney 293T (293T) cells that coexpressed FLAG-CBF-β and various ratios of two HIV-1 Vifs (HVifs): an untagged Vif and a myc-hexahistidine (HIS)-tagged (hereafter referred to as MH-tagged) Vif (Fig. 2). The FLAG-CBF-β and HVif-MH complexes were pulled down with anti-FLAG-tag beads (first co-IP), and then, the complexes in the first co-IP fraction were subjected to co-IP with an anti-HIS antibody (second co-IP). The protein contents in the co-IP complexes were compared via immunoblot analysis of the wild-type (WT) HVif and the HVif AALA mutant, in which three prolines in the PPLP were substituted with alanines. As shown in Fig. 2, the amounts of WT HVif with or without the MH tag (lanes 3, 4, and 5) varied with respect to the transfected plasmid ratio, although the amount of the MH-tagged AALA mutant was lower than that of the MH-tagged WT (lanes 6, 7, and 8). The first co-IP complexes contained the WT and HVif AALA proteins at ratios similar to those in the total lysate (α-HVif, lanes 11–16). In contrast, only negligible amounts of endogenous CBF-β were detected in the first co-IP complexes (α-CBF-β, lanes 11–16) compared with that in the total lysate (α-CBF-β, lanes 3–8). Additionally, in the second co-IP complexes, only small amounts of untagged WT (α-HVif, lanes 19 and 20) and AALA mutant (α-HVif, lanes 22 and 23) proteins were detected. These results suggest that stable Vif multimers did not form in cells.

### Identification of the minimal required Vif sequence downstream of the PPLP motif

**Fig 3:**
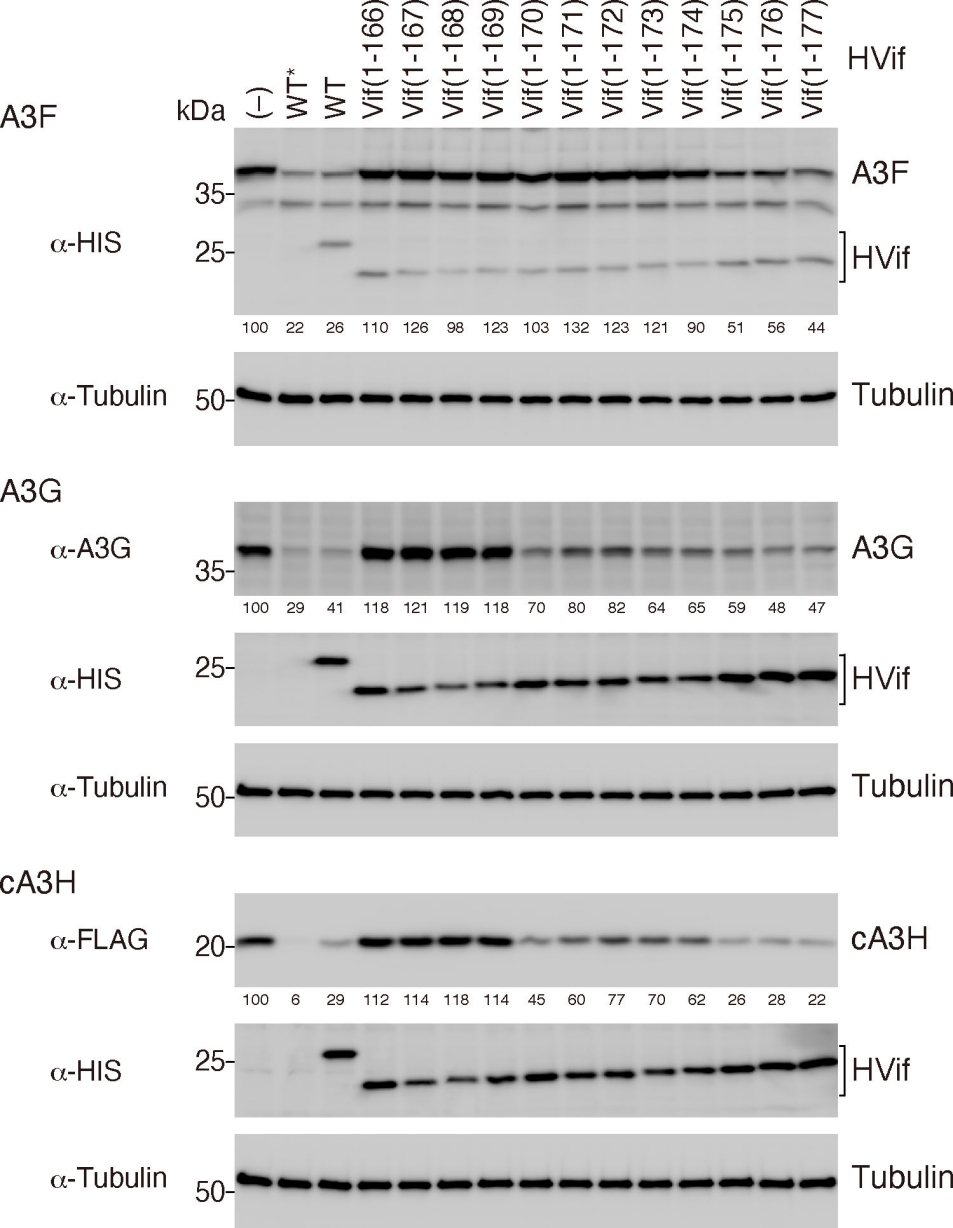
Effects of HIV-1 Vif C-terminal truncation on A3F, A3G, and cA3H degradation. A series of HVif mutants with C-terminal truncations (and a C-terminal MH tag) were coexpressed with A3F (+MH tag), A3G (no tag), or cA3H (+FLAG tag) in 293T cells. The intracellular amounts of the A3 proteins were analyzed by immunoblotting with the following antibodies: anti-HIS-tag mAb for A3F and HVif; anti-C-17 rabbit serum for A3G; anti-FLAG mAb for cA3H; and anti-β tubulin rabbit serum. (–) and WT* indicate no Vif and HVif (no tag), respectively. The percentage of A3 in the presence of HVif relative to that in the absence of HVif was calculated using the immunoblot data and is shown under each A3 image.

We next set out to determine the minimal essential sequence downstream of PPLP by constructing a series of C-terminally truncated HVif mutants. Each HVif mutant containing an MH tag at the C-terminus was coexpressed in 293T cells with A3F, A3G, or cA3H. cA3H was used in this study because it was previously reported that cA3H is more sensitive to HIV-1 NL4-3 Vif than human A3H haplotype II (32). Here, the amount of intracellular A3 was analyzed by immunoblotting (Fig. 3). As shown in the top panel, the level of A3F was lower in the presence of WT HVif than in the absence of HVif, and the reduction in the level was similar with WT HVif or without HVif carrying no C-terminal MH tag. Notably, the abilities of the mutant HVifs comprising residues 1–166 [HVif(1-166)] through 1–174 [HVif(1-174)] to reduce intracellular A3F were abolished. In contrast, HVif(1-175), HVif(1-176), and HVif(1-177) decreased the amount of A3F in these cells, although the reduction in the level was not completely comparable with that of the WT HVif. These results suggest that the minimal HVif fragment needed for A3F degradation is residues 1–175, which includes the entire F3 box required for HIV-1 Vif-mediated A3F antagonism, as reported previously (11). Moreover, HVif(1-166), HVif(1-167), HVif(1-168), and HVif(1-169) did not obviously increase A3G degradation, whereas truncated mutants with lengths longer than that of HVif(1-169) efficiently reduced the cellular A3G level. Similarly, the cA3H level decreased in the presence of WT HVif and the truncated HVif mutants that are longer than HVif(1-169). When untagged HVif was used, one additional residue (residue 171) was required for the efficient degradation of A3G and cA3H (Fig. S1). This difference in the protein length requirement might be due to the addition of the C-terminal tag onto the HVif mutant, which might stabilize the local structure of the C-terminally truncated end. These results suggest that both the PPLP motif and its downstream residues (residues 165–171) comprise the smallest fragment required for Vif-mediated A3 degradation and that the additional residues in the Vif F3 box (residues 171–175) are necessary for A3F degradation.

**Fig 4:**
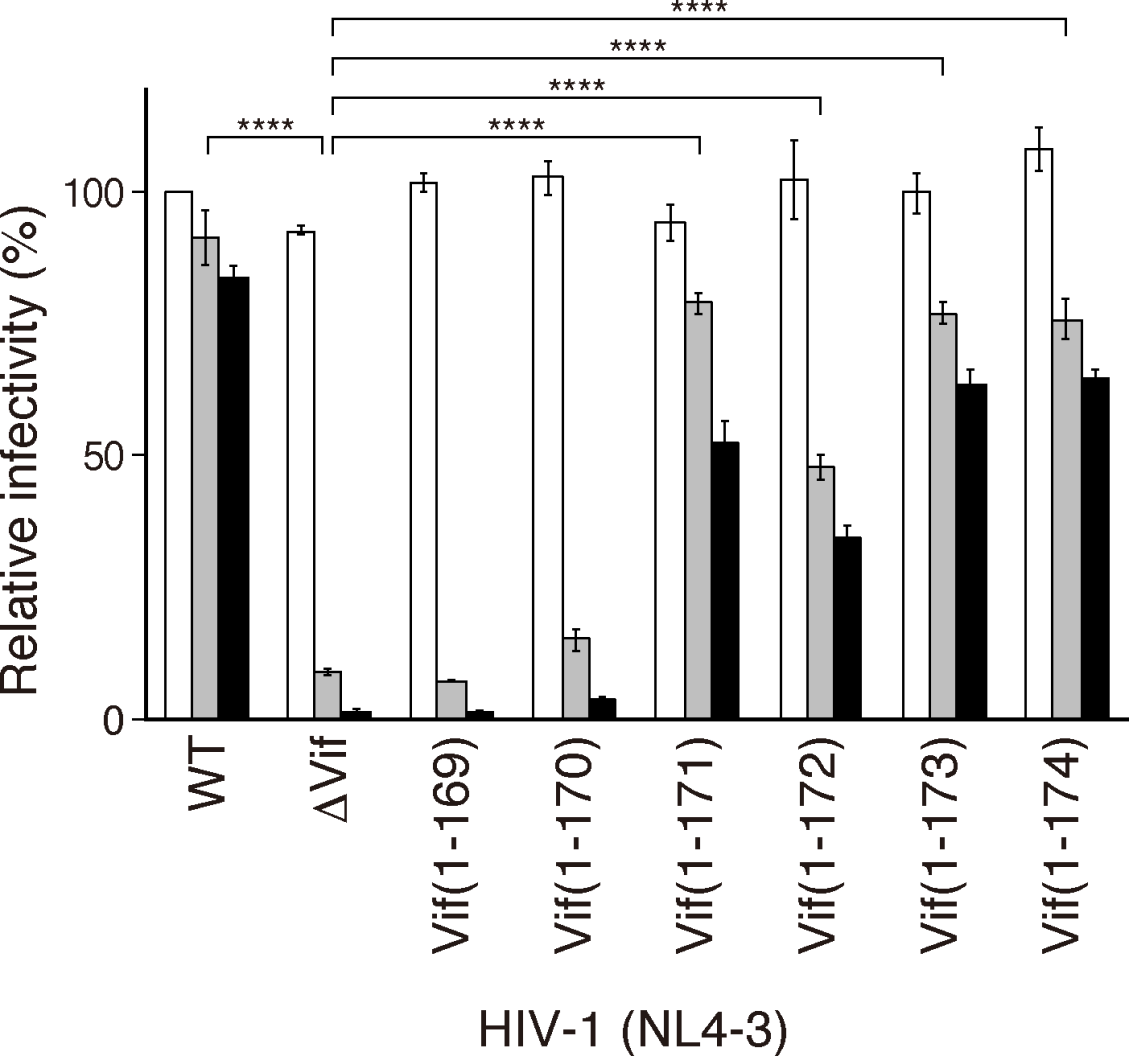
Effects of Vif truncation on HIV-1 infectivity in the presence of A3G. 293T cells were cotransfected with the A3G expression plasmid and a proviral construct (1 µg): pNL4-3 WT, pNL4-3 ΔVif, or each pNL4-3 carrying a Vif truncation mutation. The virus-containing supernatants were used to assess viral infectivity in TZM-bl indicator cells. The averages of triplicate assays are shown as the percent infectivity relative to the control (WT NL4-3 without A3G). The white, gray, and black bars represent the relative infectivity with increasing concentrations of the A3G plasmid (0 ng, 100 ng, and 500 ng, respectively). The bar graphs are representative of three independent experiments that produced similar results. The error bars represent ± the SEM from triplicate samples. Statistical significances of infectivity among the Vif variants were evaluated by parametric multiple comparison tests using PRISM9 (GraphPad). With 100 ng of the A3G plasmid (gray bars), WT, Vif(1-171), Vif(1-172), Vif(1-173), and Vif(1-174) represent significant differences in infectivity (****, *P* < 0.0001) relative to ∆Vif, but not Vif(1-169) and Vif(1-170).

Next, we assessed how the truncated mutants affected viral infectivity in the presence of A3G. We introduced a premature stop codon at the Vif coding region in HIV-1 proviral DNA by site-directed mutagenesis, cotransfected the proviral DNA along with increasing amounts of the A3G expression plasmid into 293T cells, and measured the relative infectivity using TZM-bl indicator cells. As shown in Fig. 4, the infectivity of the *vif*-deficient (∆Vif) virus decreased dramatically, whereas WT HIV-1 infectivity was only marginally affected by increased A3G expression. Similarly, the infectivity of HIV-1 expressing Vif(1-169) or Vif(1-170) was suppressed by A3G expression (gray or black columns). In contrast, the antiviral effects of A3G were attenuated with the truncated Vif mutants that were more than 170 residues long. These results also demonstrated that the short Vif fragment composed of residues 165–171 just downstream of PPLP, which forms an α-helix (α6), is the smallest fragment required for HVif to antagonize A3G-mediated anti-HIV-1 activity. Because the infectivity of the Vif(1-171)- and Vif(1-174)-expressing viruses was not fully restored to the level of the WT virus in the presence of high A3G expression (black column, Fig. 4), the residues further downstream of residue 174 might partially contribute to Vif function.

### Predicting the structural stability with molecular dynamics (MD) simulations

**Fig 5:**
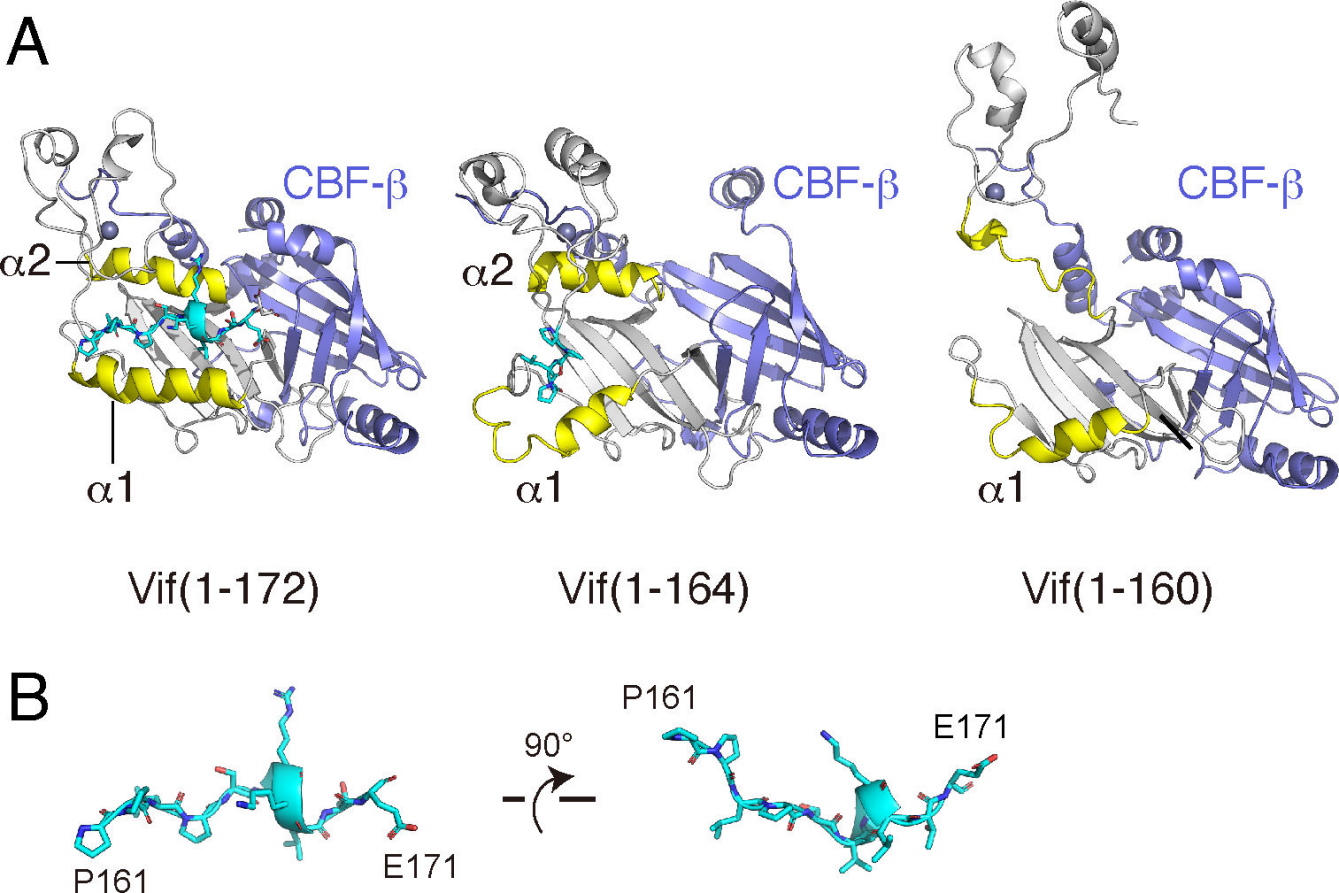
Molecular dynamics (MD) simulations of the HIV-1 Vif/CBF-β complex structure. (**A**) The structures during the 10-ns MD simulations are shown as ribbons for the truncated Vif mutants Vif(1-172) and Vif(1-160) (gray) in complex with CBF-β (violet). Vif residues 14–30 and 100–112 in helices 1 (α1) and 2 (α2) are colored yellow. The PPLP motif and its downstream region are shown in cyan, whereas the zinc ions are shown as violet spheres. (**B**) PPLP-α6 (residues 161–171) solely (cyan) is shown. This fragment adopts a stable L-like configuration.

To further understand why deletions in the PPLP-α6 fragment abolish Vif function, we performed MD simulations with Vif mutants complexed with CBF-β. In the simulations with Vif(1-172), the conformation of Vif remained constant during the 10-ns MD simulation and was similar to that in the crystal structure (Fig. 5A, left panel). In contrast, the parallel arrangement of α1 and α2 gradually became distorted in the Vif(1-164) simulation (Fig. 5A, middle panel). Similarly, the Vif conformation drastically changed during the Vif(1-160) simulation, especially the configurations of α1 and α2, which completely collapsed (Fig. 5A, right panel). These results suggest that PPLP-α6 is required for maintaining Vif structural integrity by constraining α1 and α2 in the correct position. Interestingly, the MD simulations with Vif(1-172) indicated that this undecapeptide region corresponding to PPLP-α6 (residues 161–171) solely adopts a stable L-like configuration (Fig. 5B).

### Formation of the tetrameric VCBC complex using recombinant proteins

**Fig 6:**
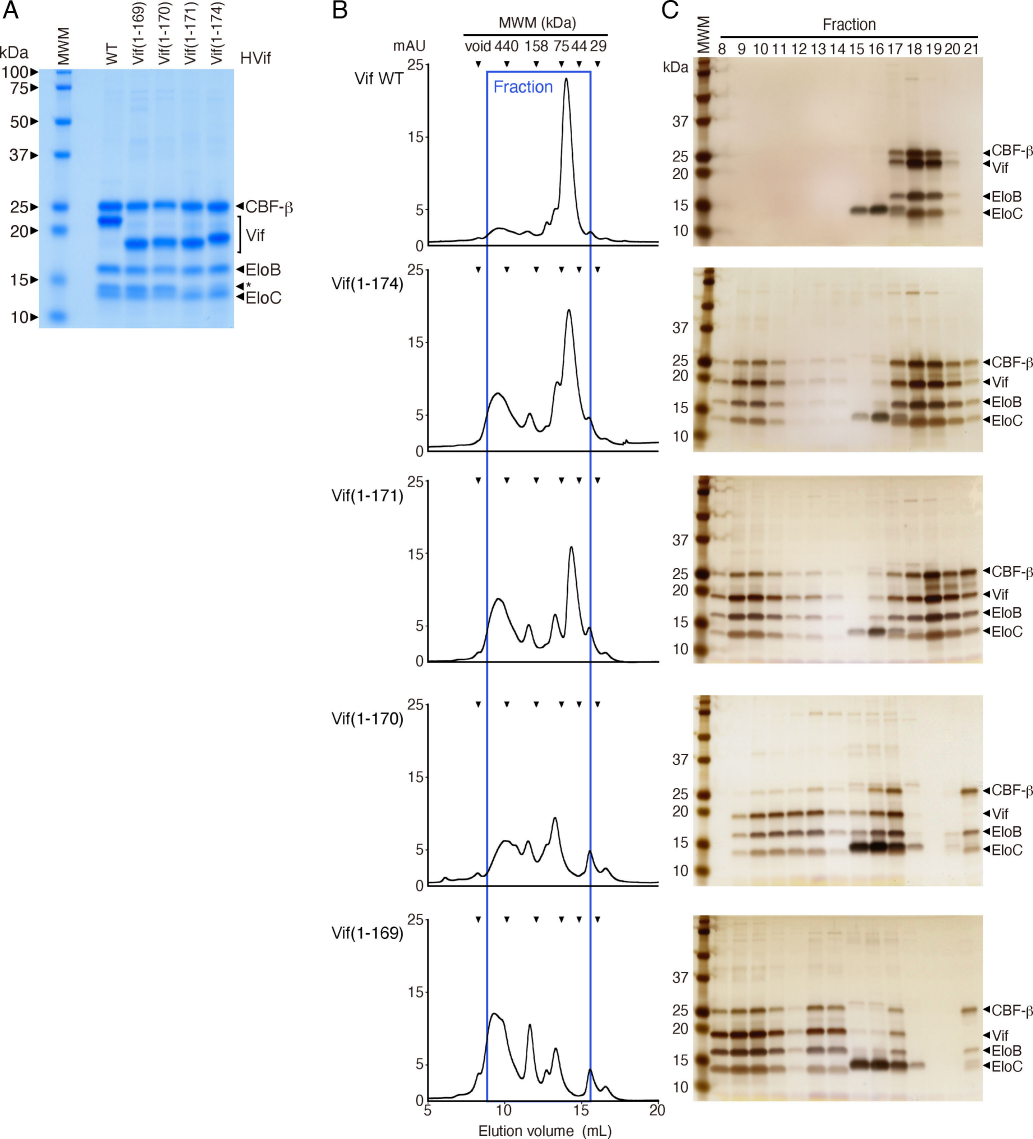
Analysis of the Vif/CBF-β/EloB/C (VCBC) tetrameric complexes after *in vitro* reconstitution using recombinant proteins. (**A**) Complexes containing WT or mutant Vif purified by cation-exchange chromatography were separated by SDS–PAGE and stained with Coomassie Brilliant Blue. (B and C) The approximate molecular sizes of the complexes were analyzed by size-exclusion chromatography. Chromatographic profiles (**B**) and silver-stained SDS–PAGE gel images (**C**) of the protein components in each fraction. From the top, the profiles of the following complexes are shown: Vif WT/HIS-CBF-β/EloB/C, Vif(1-174)/HIS-CBF-β/EloB/C, Vif(1-171)/HIS-CBF-β/EloB/C, Vif(1-170)/HIS-CBF-β/EloB/C, and Vif(1-169)/HIS-CBF-β/EloB/C. Each tetrameric complex (20 µM) was applied to a Superdex 200 increase 10/300 Gl column at a flow rate of 0.25 mL/min for FPLC analysis. The chromatography data of each fraction are given as absorbance values (mAUs) at UV 280 nm. The asterisk indicates an unknown bacterial protein. The chromatograms and SDS–PAGE gel images are representative of three independent experiments that produced similar results.

To validate the structural mechanism predicted by the MD simulations, we analyzed the stability of the VCBC complex in a reconstituted system using recombinant proteins. Since CBF-β, EloB, and EloC are known to greatly increase the solubility of full-length Vif *in vitro* (4, 33, 34), the tetrameric VCBC complexes that were coexpressed in *Escherichia coli* were purified via tandem affinity and ion-exchange chromatography. Then, the sizes of the complexes were analyzed by size-exclusion column chromatography (SEC). As shown in Fig. 6A, all the VCBC complexes containing the WT or Vif mutants, Vif(1-169), Vif(1-170), Vif(1-171), and Vif(1-174), were obtained as soluble complexes (Fig. 6A). On the basis of the intensity of each band in the SDS–polyacrylamide gel electrophoresis (PAGE) images, the ratios of the VCBC components were similar among the WT and mutant Vifs. However, SEC analysis revealed that the sizes of the tetrameric complexes composed of the functionally defective Vif(1-169) and Vif(1-170) mutants, and the proficient Vif(1-171) and Vif(1-174) mutants differed. The Vif WT complex displayed monodisperse, and its mass corresponded to a single complex unit (approximately 72.0 kDa) (Fig. 6B). In addition, after staining with Coomassie Brilliant Blue (CBB) and Silver Stain KANTO III, the ratio of Vif WT:CBF-β:EloB:EloC was estimated to be 1:1:1:1 (Fig. 6), which is consistent with the findings of a previous study (34). Similarly, the major fractions of the complexes containing the functionally proficient Vif mutants Vif(1-174) and Vif(1-171) eluted as uniform fractions with masses of approximately 66.2 kDa and 61.2 kDa, respectively (Fig. 6B). In contrast, for the tetrameric complexes containing functionally defective mutants Vif(1-170) and Vif(1-169), the peak corresponding to the Vif WT complex was diminished, and the sizes of the complexes were irregular, possibly due to the formation of aggregates with masses of approximately 400–1,000 kDa (Fig. 6B and C). Therefore, these results suggest that the fragment Vif(1-171) is sufficient to form the VCBC tetrameric complex and that PPLP-α6 plays an important role in maintaining the stable formation of this monogenous complex; moreover, these results are supported by structural analysis data of the MD simulations (Fig. 5).

### *In vitro* A3 polyubiquitination

**Fig 7:**
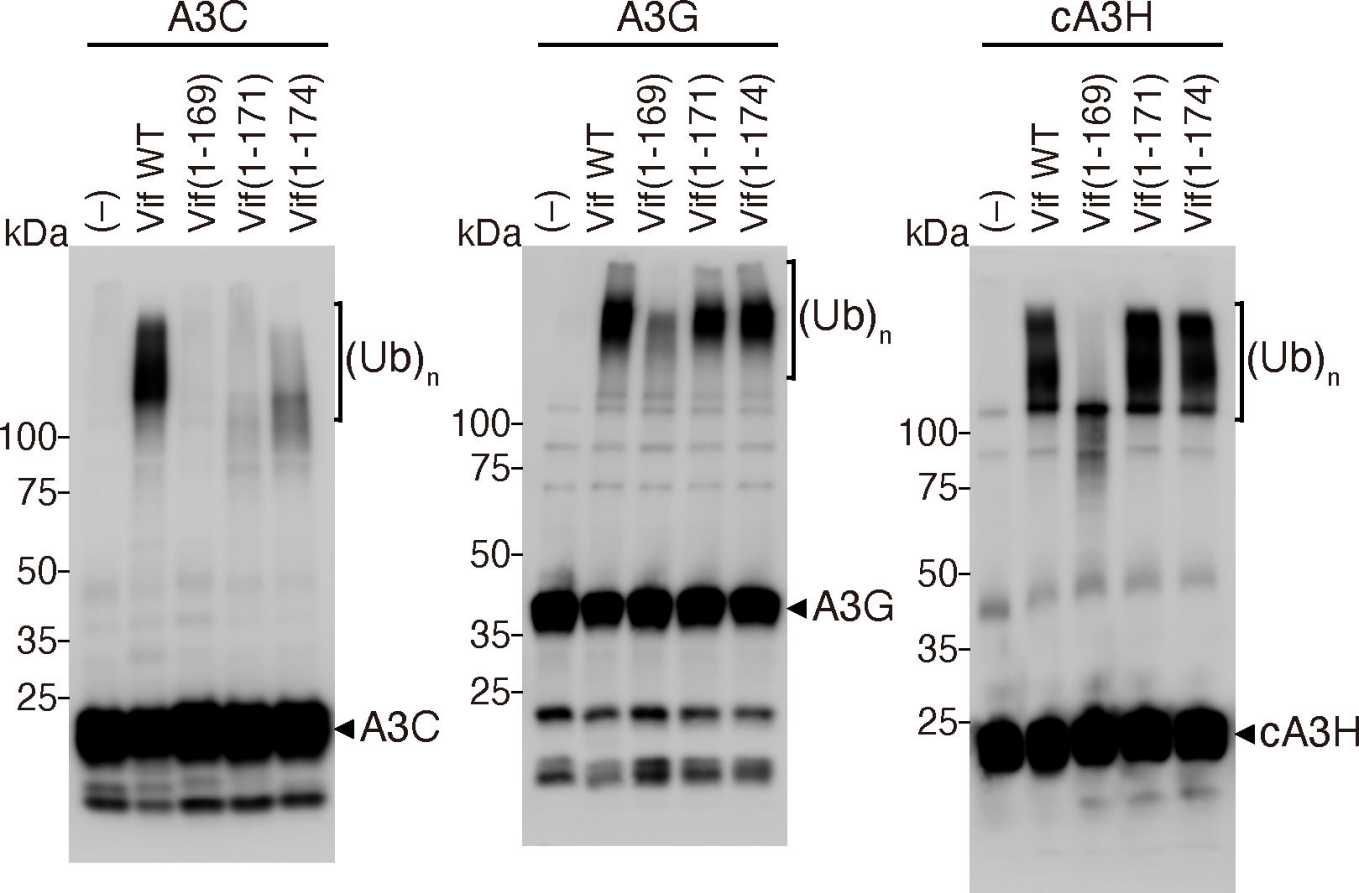
*In vitro* A3 ubiquitination with recombinant proteins and HVif mutants. The A3-MH proteins A3C, cA3H, and A3G were incubated with the Cul5/Rbx2/Vif/HIS-CBF-β/EloB/C complexes in the presence of ubiquitin (Ub), where the Vif protein varied: Vif WT, Vif(1-169), Vif(1-171), or Vif(1-174). A3s were separated by SDS–PAGE and visualized by immunoblotting using the following antibodies: an anti-Myc-Tag mAb for A3C and cA3H; and anti-C-17 rabbit serum for A3G. (Ub)_n_ represents polyubiquitinated products. All experiments were repeated three times and produced equivalent results.

To further evaluate Vif function, we performed an *in vitro* reconstituted ubiquitination assay using WT and mutant Vif proteins as previously described (4, 5, 35). We selected three A3 proteins, MH-tagged A3C, A3G, and MH-tagged cA3H, which interact with different Vif surface regions. In the absence of the tetrameric VCBC complex, no A3 polyubiquitin products were detected (Fig. 7). With WT Vif, polyubiquitinated A3s, which appeared as blurry bands with high molecular weights, were detected. As expected, in the presence of Vif(1-171) or Vif(1-174), polyubiquitinated A3G and cA3H were also detected. In contrast, the amounts of A3 polyubiquitination products were dramatically reduced with the Vif(1-169) mutant. Notably, because the Vif F3 box (residues 171–175) is part of the Vif interface responsible for the A3C/A3F interaction (11), no significant polyubiquitination of A3C was observed in the presence of the Vif(1-171) and Vif(1-174) complexes (Fig. 7, left). These results revealed that Vif(1-171) has sufficient length to function as an adaptor for Vif-mediated ubiquitination *in vitro*, which coincides with the results from the cell culture assays (Fig. 3 and 4), MD simulations (Fig. 5), and VCBC complex analysis (Fig. 6).

### Mutational analysis of the hydrophobic residues in PPLP-α6

**Fig 8:**
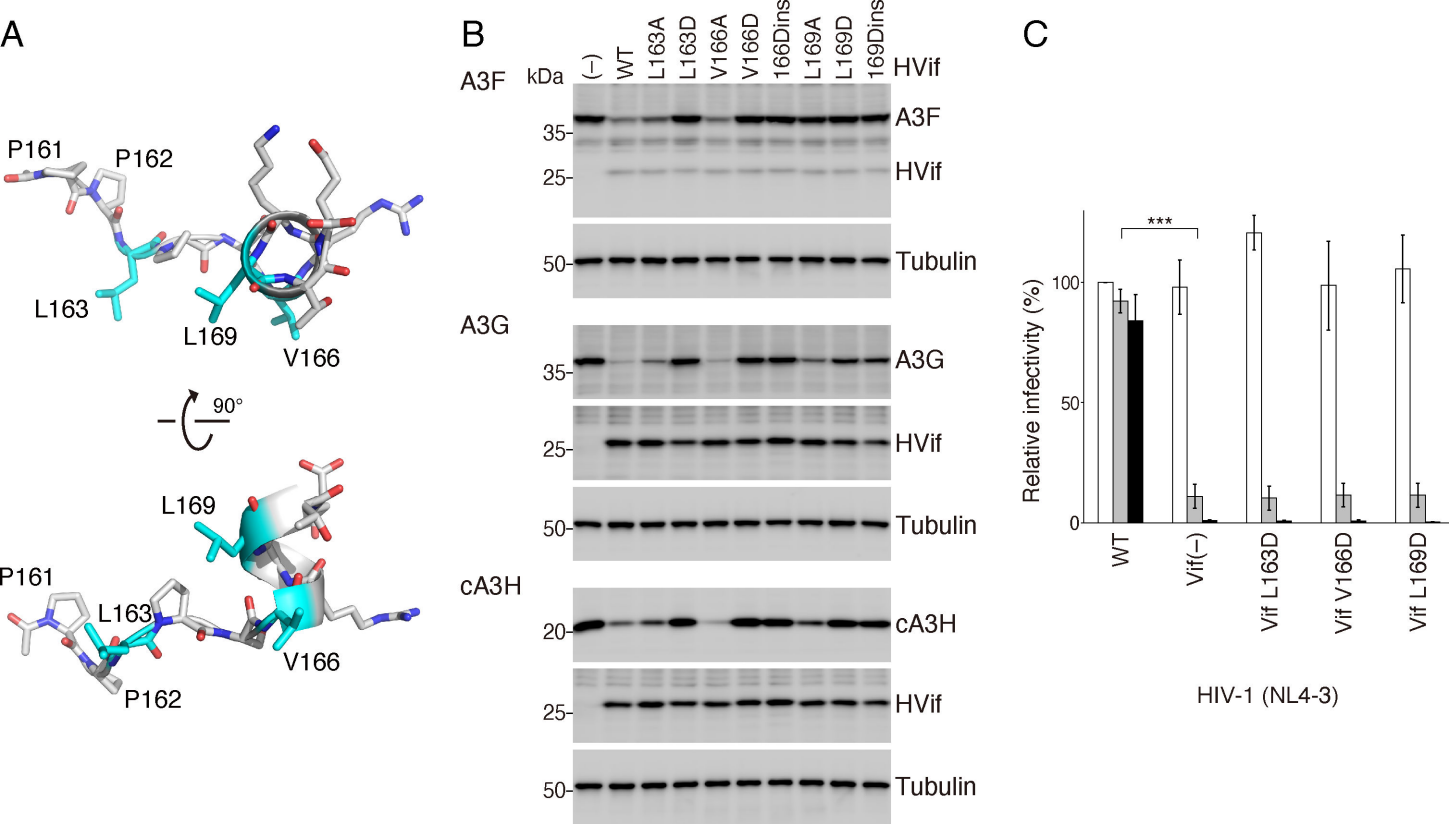
Functional analysis of the region downstream of the PPLP motif. (**A**) Ribbon representation of the PPLP motif (residues 161–164) and its downstream short α-helix (residues 165–171). The ring on P164 and the hydrophobic side chains of L163, V166, and L169, shown in cyan, face the same side. (**B**) Effect of mutation to each hydrophobic residue on A3 degradation. The WT or mutant Vifs carrying an MH tag were coexpressed with A3F, A3G, or cA3H in 293T cells, and the intracellular protein levels of A3s were analyzed by immunoblotting. HVif 166Dins and HVif 169Dins carry an aspartate residue insertion at positions 166 and 169, respectively. (**C**) Effect of the *vif* mutation on HIV-1 infectivity in the presence of A3G. 293T cells were cotransfected with the A3G expression plasmid and a proviral construct (1 µg), pNL4-3 WT, pNL4-3 ΔVif, or each pNL4-3 carrying the indicated *vif* mutation. Viral infectivity was analyzed by TZM-bl indicator cell assays. The averages of triplicate assays are shown as the percent infectivity relative to the control (WT NL4-3 without A3G). The white, gray, and black bars represent the relative infectivity with increasing concentrations of the A3G plasmid (0 ng, 100 ng, and 500 ng, respectively). The error bars represent ± the SEM from Triplicate samples. Statistical significances of infectivity among the vif variants were evaluated by parametric multiple comparison tests. In the presence of 100 ng of the A3G plasmid (gray bars), WT show significantly different infectivity relative to Vif(–) (***, *P* < 0.001), but not Vif L163D, Vif V166D, and Vif L169D.

Our MD simulations suggested that PPLP-α6 allows α1 and α2 to adopt stable conformations (Fig. 5). Importantly, the hydrophobic side chains of L163, V166, and L169, which are highly conserved among HIV-1/SIVcpz Vifs (Fig. 1), project inward (Fig. 8A). Therefore, we assumed that these hydrophobic residues are also important for stabilizing the local conformation of Vif. To examine this possibility, we investigated how mutating Vif L163, V166, or L169 affected A3 antagonism. A3F, A3G, or cA3H were coexpressed with the HVif mutants in 293T cells, and the intracellular levels of A3s were compared by immunoblotting. The results revealed that the HVif mutants L163A and V166A reduced the intracellular levels of A3F, A3G, and cA3H, whereas L169A decreased the levels of A3G and cA3H but not A3F (Fig. 8B). In contrast, single mutations of these residues to aspartate (L163D, V166D, and L169D) abolished the Vif-mediated degradation of A3F, A3G, and cA3H. In addition, mutants HVif 166Dins and HVif 169Dins, which carry one-residue insertions that may disrupt the helical structure of PPLP-α6, were also unable to degrade A3F, A3G, and cA3H.

We further analyzed the effects of mutating the hydrophobic residues in PPLP-α6 on viral infectivity in the presence of A3G (Fig. 8C). Without A3G, the mutant HIV-1 (L163D, V166D, and L169D) displayed high infectivity that was similar to that of the WT. In contrast, the infectivity of the HIV-1 Vif L163D, V166D, and L169D mutants drastically decreased in the presence of A3G and was similar to that of ∆Vif HIV-1. These results demonstrate that the three hydrophobic residues, L163, V166, and L169, in PPLP-α6 are important for A3G antagonism, which is consistent with the effects on A3G protein degradation.

## DISCUSSION

**Fig 9:**
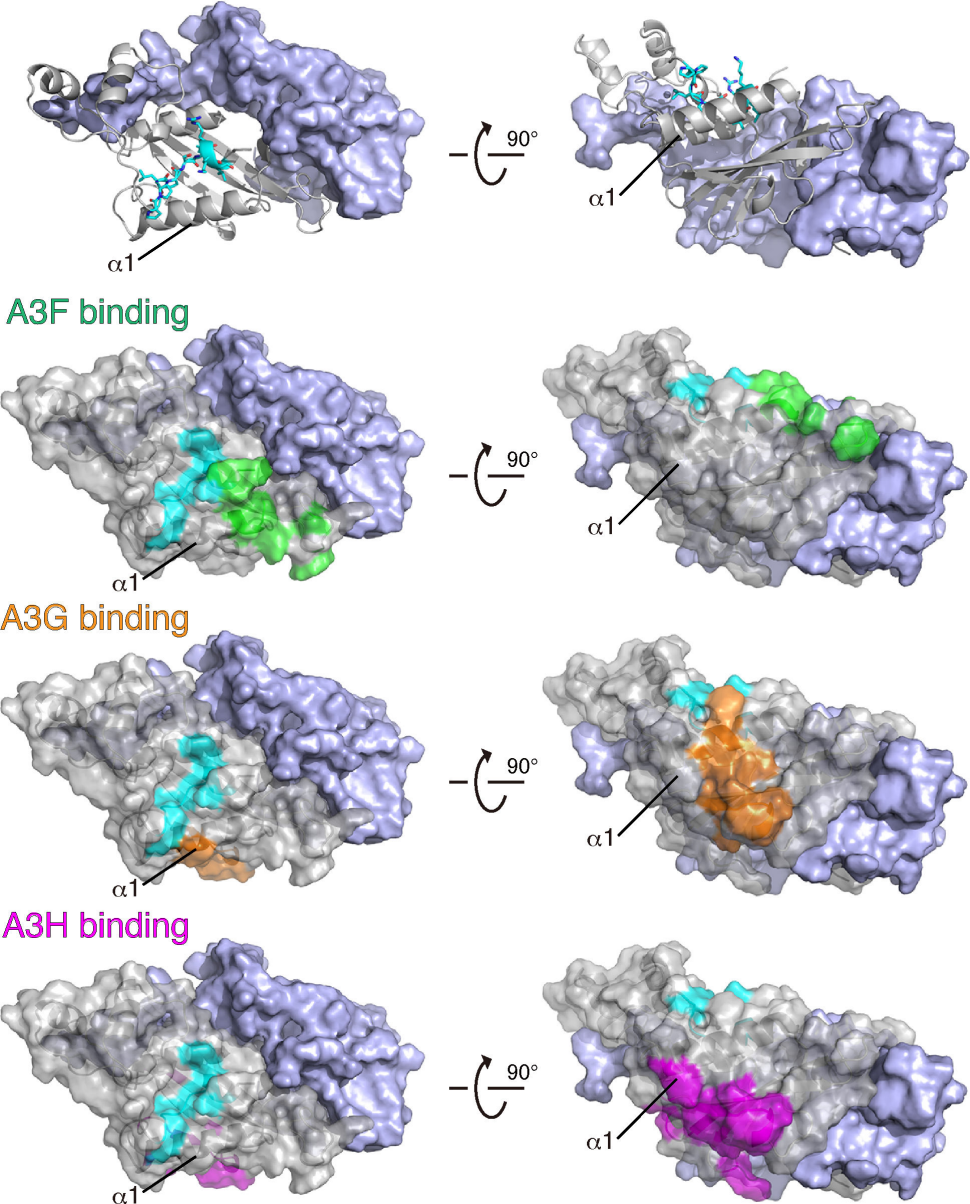
The PPLP motif and other residues responsible for A3 binding to the Vif/CBF-β complex. On top, the ribbon and cyan surface representations represent HIV-1 Vif(1-174) and CBF-β, respectively. The PPLP motif is colored light blue. The Vif residues critical for A3F, A3G, and A3H binding are colored green, orange, and magenta, respectively. α1 neighboring the PPLP motif is located close to these A3 interaction regions.

A unique tetrapeptide motif, PPLP, which is highly conserved among HIV-1 lineage Vifs, is known as a critical sequence for Vif function in virus replication, although the mechanism remains unclear. In this study, we identified an essential Vif region, PPLP-α6, which consists of the PPLP motif and its immediate downstream sequence α6, as being crucial for antagonizing the antiviral effects of A3F, A3G, and cA3H (Fig. 3 and 4). MD simulations of the Vif/CBF-β complex suggested that PPLP-α6 maintains the structural integrity of Vif by binding to α1 and α2 (Fig. 5). Because α1 predominantly includes interface residues critical for interactions with A3s (Fig. 9), PPLP-α6 plays a critical role in maintaining the conformation of the A3-binding interfaces. Our *in vitro* ubiquitination experiments using recombinant proteins demonstrated that PPLP-α6 was required for the formation of stable, monogenous VCBC tetramers (Fig. 6) and efficient A3 polyubiquitination (Fig. 7). Three hydrophobic residues in PPLP-α6, L163, V166, and L169, which are highly conserved among HIV-1 Vifs, are important for A3G antagonism and degradation (Fig. 1 and 8).

It is currently believed that the PPLP motif may play a critical role in Vif homomultimerization (28–30). However, our co-IP assays (Fig. 2) and SEC analysis (Fig. 6) showed no evidence of the formation of stable Vif oligomers. In addition, Vif oligomers have not been observed in any of the structures of the VCBC and A3-VCBC complexes determined by X-ray crystallography or cryo-EM (3, 10, 14–16). Because the earlier evidence of Vif multimerization via PPLP was obtained from *in vitro* experiments using the recombinant Vif protein only but not the CBF-β scaffold protein, the effects of the PPLP motif on Vif oligomerization might have not been appropriately assessed due to the possibility that the Vif proteins were misfolded, such as the observed aggregation of Vif when this protein was overexpressed alone. Therefore, we propose that the Vif PPLP sequence is not the homomultimerization motif crucial for viral replication.

In the reported VCBC complex structures (10, 14–16), PPLP-α6 forms a kink that immediately follows the short helix α6 and PPLP appears to contribute to nucleate folding and stabilization of the short downstream helix α6. Additionally, the reported structures show that residues P161 and P164 interact with residues H27 and H28 in α1 via CH–π interactions inside the Vif molecule (3, 10, 14–16). Residues H27 and H28 are highly conserved in HIV-1 lineage Vifs. This notion coincides with a previous report demonstrating that double mutation to H27A-H28A severely hampered the interaction between Vif and CBF-β, causing significant defects in Vif-mediated A3G degradation (36). These unique structural features of the rigid PPLP-α6 segment are most likely involved in maintaining the integrity of the local conformation to provide interaction interfaces for the three types of A3 family proteins. Therefore, it is rationalized that the minimal required length of Vif determined in this study was the sequence to the end of α6 (Fig. 6). Interestingly, only residues 1–172 of Vif have been assigned in the X-ray crystal structure of the pentameric VCBC/N-terminal Cul5 complex (3). As this crystal structure was determined after the pentameric protein complex was treated with elastase, the C-terminal Vif region downstream of α6 might be flexible, whereas PPLP-α6 is rigid.

Besides consecutive proline residues, hydrophobic residue repeats at L163, V(or I)166 and L169, and S165 in the PPLP-α6 sequence are highly conserved among the HIV-1 lineage Vifs. In addition, since proline sterically restricts the backbone conformation of neighboring residues, the PPLP-α6 region is considered to adopt a rigid local conformation. Interestingly, according to the structure of the VCBC complex, PPLP-α6 also contributes to the formation of a deep hydrophobic pocket in the Vif molecule (3). Because mutations to the critical residues of PPLP-α6 severely disrupted Vif-mediated antagonism of all three types of A3s (Fig. 8), this pocket could be a very attractive target for the design of small compound inhibitors against HIV-1 Vif. Indeed, the Vif mutants in which PPLP was changed to AALA or deleted displayed a dominant negative effect on the ability of WT Vif to antagonize A3G in cells and virions (26, 37, 38). Moreover, Bennett et al reported that camptothecin (CPT)-derived analogs that are inactive against Topoisomerase I block the Vif-mediated degradation of A3F and A3G, thereby suppressing HIV-1 replication in A3F/G-expressing CD4-positive T cells (39). Molecular modeling studies predicted that the CPT analogs could bind to the PPLP-α6 pocket. Although accommodation of the CPT-derived molecule in this pocket has not been confirmed by structural analyses, such as cocrystal analysis, this is important evidence that is needed to explore Vif inhibitors that target the conserved and rigid pocket at PPLP-α6.

In summary, we herein elucidated the functional roles of the unique HIV-1 PPLP motif. Although PPLP may play a critical role in Vif homomultimerization, our reassessment revealed no strong evidence of Vif multimerization via the PPLP motif. Rather, we found that PPLP, in conjunction with its short downstream segment α6, plays a crucial role in antagonizing the antiviral effects of A3F, A3G, and cA3H. In the Vif structure, PPLP-α6 contributes to fixing the local Vif interface conformation so that three different types of A3 proteins can be recognized. Hence, these results suggest that unique PPLP-α6 plays critical allosteric roles in maintaining the integrity of the A3 recognition interfaces. The findings here provide important evidence for understanding the functions of HIV-1 Vif as well as for the design of novel antiviral strategies targeting HIV-1 Vif, which could possibly be achieved by disrupting the PPLP-mediated stabilization of the A3 recognition interfaces.

## MATERIALS AND METHODS

### Cell culture

293T and TZM-bl cells were maintained in Dulbecco’s modified Eagle medium (Sigma–Aldrich) supplemented with 10% fetal bovine serum, penicillin (100 U/mL), and streptomycin (100 µg/mL) (Thermo Fisher Scientific) in 5% CO_2_ at 37°C. TZM-bl cells were obtained from the AIDS Research and Reference Reagent Program, NIAID, NIH (40–43).

### Plasmids

Mammalian expression plasmids for A3F (MH), A3G (MH), and cA3H (FLAG) were used as previously described (11, 32). For the CBF-β expression plasmid pcDNA FLAG-CBF-β, the cDNA fragment of isoform 2 (GenBank ID NM_001755) was amplified by nested reverse transcription–PCR (RT–PCR) from total RNA from healthy-donor peripheral blood mononuclear cells using the PrimeScript II High Fidelity One-Step RT–PCR Kit (Takara Bio); the primer sets used are listed in Table S1. The CBF-β cDNA fragment was inserted into pcDNA3.1(–) between the NotI and EcoRI sites. The Vif expression plasmid with a codon-optimized *vif* ORF fragment derived from an HIV-1 NL4-3 was used as previously described (44). For each Vif mutant, single premature stop codon mutations or substitution mutations were introduced by oligonucleotide-directed PCR using appropriate primer sets (Table S1).

For the bacterial expression plasmid pETDuet Vif/HIS-CBF-β, the *cbf-*β gene fragment containing the N-terminal HIS tag-coding sequence was ligated into multiple cloning site (MCS)−1 of the pETDuet-1 vector (Merck Millipore), and subsequently, the *vif* fragment that was amplified by PCR using appropriate primer sets (Table S1) was inserted into MCS-2 at the NdeI and XhoI sites. For pRSFDuet HIS-Cul5/Rbx2, DNA fragments of the *cul5* (residues 1–780, GenBank ID NM_003478) and *rbx2* genes (residues 1–113, GenBank ID NM_014245) were amplified by PCR using primers containing restriction site sequences (Table S1). The PCR product of Cul5 containing an N-terminal HIS tag was digested with two restriction enzymes, BamHI and NotI, and ligated into MCS-1 of pRSFDuet-1 (Merck Millipore). The *rbx2* gene product, digested with NdeI and BglII, was then ligated into MCS-2 of pRSFDuet HIS-Cul5. The *rbx2* gene fragment was also ligated into the pET-21a(+) plasmid (Merck Millipore) at the NdeI and NotI sites. To construct the pEU-E01-A3C-MH and pEU-E01-cA3H-MH plasmids for wheat germ cell-free protein synthesis (45, 46), each A3 DNA fragment with a tobacco etch virus protease (TEV) cleavage site followed by a C-terminal MH tag was amplified by PCR using two primer sets each (Table S1) and then ligated into pEU-E01-MCS-TEV-HIS C1 (Cell-Free Sciences).

### Transfection, co-IP, and immunoblotting

To analyze the protein levels in eukaryotic cells, the A3 and Vif expression plasmids were cotransfected into 293T cells using FuGENE HD (Promega), and the protein levels were analyzed by immunoblotting as previously reported (47). Additionally, anti-HIS tag monoclonal antibody (mAb) (1:2,500 dilution; Medical & Biological Laboratories Co.), anti-CBFb rabbit mAb (EPR6322) (1:10^3^ dilution; Abcam), and goat horseradish peroxidase-conjugated secondary antibodies against mouse IgG (1:2 × 10^4^ dilution) or rabbit IgG (1:2 × 10^4^ dilution) (Thermo Fisher Scientific) were used.

In the two-step co-IP assay, HVif WT or HVif AALA mutants carrying a C-terminal MH tag were coexpressed with untagged WT HVif and FLAG-CBF-β in 293T cells. The transfected cells were lysed in co-IP lysis buffer (50 mM Tris HCl [pH 7.4], 150 mM NaCl, 1 mM EDTA, 1% Triton X-100, and 1 µg/mL RNase A) on ice for 30 min. The soluble fraction of the lysate was cleared by centrifugation at 20,000 × *g* for 30 min at 4°C. Anti-FLAG M2 magnetic beads (40 µL/mL) (Sigma–Aldrich) were added to the cleared lysate for 30 min of incubation on ice, and then, the sample was washed with co-IP buffer five times. The protein complex bound to the beads was eluted with 3× FLAG peptide (300 ng/µL) (Sigma–Aldrich) in co-IP elution buffer (50 mM Tris HCl [pH 7.4], 150 mM NaCl and 1 mM EDTA). One-fourth of the eluted sample was used for immunoblotting as the first co-IP fraction. The remaining sample was diluted 3-fold and subjected to a second co-IP using an anti-HIS rabbit polyclonal antibody (4 µL/sample) (Medical & Biological Laboratories Co., cat# PM032) and Dynabeads protein G (30 µL/mL) (Thermo Fisher Scientific). The second co-IP complex bound to the beads was washed with co-IP buffer five times and then dissolved in Laemmli buffer (Bio-Rad) containing 2.5% 2-mercaptoethanol (2-ME) for immunoblotting.

### Infectivity assay

The effects of the *vif* mutations on HIV-1 infectivity in the presence of A3G were analyzed in TZM-bl cells as previously described (11).

### Protein expression and purification of the Vif/HIS-CBF-β/EloB/C complex

The Vif/HIS-CBF-β/EloB/C complex expressed in *E. coli* was purified according to previously reported protocols with slight modifications (3–5). Briefly, two plasmids, pETDuet Vif/HIS-CBF-β and pRSFDuet EloB/C, were cotransformed into BL21 Star (DE3) competent cells (Thermo Fisher Scientific). The transformed bacteria were grown in Luria–Bertani (LB) media supplemented with ampicillin (75 µg/mL) and kanamycin (50 µg/mL) at 37°C to an optical density of ~0.6 at 600 nm. After being chilled to ~20°C on ice, the bacteria were further cultured with 0.5 mM isopropyl β-D-1-thiogalactopyranoside (IPTG) and 10 µM ZnSO_4_ at 20°C for 20 h. The bacterial pellets were harvested, resuspended in lysis buffer I (50 mM Tris HCl [pH 8.0], 500 mM NaCl, and 5 mM 2-ME), and sonicated on ice. After adding one-third the volume of lysis buffer II (50 mM Tris HCl [pH 8.0], 500 mM NaCl, 5 mM 2-ME and 3% Triton X-100), the bacterial suspensions were incubated for 30 min at 4°C and then subjected to centrifugation at 23,000 × *g* for 30 min at 4°C, followed by filtration through a 0.8 µm pore-size membrane. The cleared soluble fraction was applied to a Ni-Sepharose 6 Fast Flow column (Cytiva) for affinity purification. The column was washed with 40 column volumes of imidazole wash buffer (50 mM Tris HCl [pH 8.0], 500 mM NaCl and 40 mM imidazole). The Vif/HIS-CBF-β/EloB/C complex fraction was obtained using imidazole elution buffer (50 mM Tris HCl [pH 8.0], 500 mM NaCl, 300 mM imidazole and 5 mM 2-ME). The elute was subsequently purified through a two-step method of cation-exchange chromatography using Q Sepharose High Performance (Cytiva) in a fast protein liquid chromatography (FPLC) system (ÄKTA pure; GE Healthcare). The equilibration, wash, and elution buffers contained 25 mM Tris HCl (pH 8.0) and 5 mM 2-ME in addition to 25 mM NaCl, 200 mM NaCl, and 500 mM NaCl, respectively. The eluted complex was further purified by gel filtration using a HiLoad 26/600 Superdex 200 prep grade column (Cytiva) in FPLC, concentrated to 40 µM with a Centriprep 10K (Merck Millipore), and then stored at –80°C prior to use.

### Protein expression and purification of the Cul5/Rbx2 complex

The HIS-Cul5/Rbx2 protein complex expressed in *E. coli* was purified according to previous reports with minor modifications (4, 5). Briefly, two plasmids, pRSFDuet HIS-Cul5/Rbx2 and pET Rbx2, were used to express an excess of the Rbx2 protein because insufficient expression of Rbx2 was observed in *E. coli* transformed with pRSFDuet HIS-Cul5/Rbx2 only. Similar to the protocol for Vif/HIS-CBF-β/EloB/C expression, BL21 Star (DE3) *E. coli* transformed with the two plasmids were grown in LB media containing ampicillin (75 µg/mL) and kanamycin (50 µg/mL) at 37°C, cooled to ~16°C on ice and induced with 0.5 mM IPTG and 10 µM ZnSO_4_ at 16°C for 20 h. The bacteria were resuspended in lysis buffer I and sonicated, followed by incubation for 30 min on ice after the addition of lysis buffer II. Next, the lysate was cleared by centrifugation and membrane filtration. The cleared fraction was subjected to affinity chromatography using a Ni-Sepharose 6 Fast Flow column. The column was washed with 40 column volumes of imidazole wash buffer, and the HIS-Cul5/Rbx2 complex was eluted with imidazole elution buffer. The eluted fraction was further purified by gel filtration using the Superdex 200 gel column in FPLC. The fraction containing protein that was the appropriate size for the HIS-Cul5/Rbx2 complex was collected and concentrated to approximately 40 µM with a Centriprep 30K (Merck Millipore). The purified HIS-Cul5/Rbx2 protein complex was stored at –80°C prior to use.

### Expression and purification of the A3 proteins

To express the recombinant proteins A3C-MH and cA3H-MH, *in vitro* transcription and wheat germ cell-free protein synthesis (45, 46) were performed using the WEPRO7240H expression kit (Cell-Free Sciences) according to the manufacturer’s instructions. Briefly, each A3 mRNA was transcribed *in vitro* from the plasmid DNA templates with SP6 RNA polymerase. *In vitro* translation was performed in bilayer mode, in which the bottom layer was supplemented with 40 µM ZnSO_4_. Each A3 translation product was resuspended in an equal volume of A3 lysis buffer (50 mM Tris HCl [pH 8.0], 1 M NaCl, 5 mM 2-ME, 40 mM imidazole, 1% Triton X-100, and 50 ng/µL RNase A) and then incubated for 1 h at 4°C. The suspensions were subjected to centrifugation at 10,900 × *g* for 20 min at 4°C. For purification of the MH-tagged A3s, the soluble fraction was subjected to a Ni-Sepharose High Performance column for affinity purification via the batch method. The resins were washed five times with A3 wash buffer (50 mM Tris HCl [pH 8.0], 500 mM NaCl, 0.5% Triton X-100, and 40 mM imidazole), and the A3 proteins were then eluted with A3 elution buffer (50 mM Tris HCl [pH 8.0], 500 mM NaCl, 5 mM 2-ME, 0.5% Triton X-100, and 300 mM imidazole). The recombinant untagged A3G protein was expressed in a baculovirus expression system and purified as previously described (48). The purities of the A3 proteins were confirmed by SDS–PAGE followed by CBB staining, and the concentrations were determined by absorbance measurements at UV_280_. The purified A3 proteins were stored at –80°C before use.

### Analytical size-exclusion chromatography

Each HIS-CBF-β/EloB/C complex with WT or mutant Vif (20 µM) in gel filtration elution buffer (25 mM Tris HCl [pH 8.0], 500 mM NaCl, and 5 mM 2-ME) was injected onto a Superdex 200 Increase 10/300 Gl column (Cytiva) at a flow rate of 0.25 mL/min for FPLC analysis, and the UV absorbance was monitored at 280 nm. The column was calibrated using a gel filtration calibration kit (Cytiva) consisting of the following: blue dextran (void volume), ferritin (440 kDa), aldolase (158 kDa), conalbumin (75 kDa), ovalbumin (44 kDa), and carbonic anhydrase (29 kDa). Each 0.5 mL fraction around the peak of interest was concentrated by ultrafiltration using Amicon Ultra 0.5 mL filters (MWCO 10 kDa) (Merck Millipore). The concentrated proteins were separated on an 8%–16% gradient Mini-PROTEAN TGX gel (Bio-Rad) and stained with SimplyBlue Safe stain (Thermo Fisher Scientific) for CBB staining or Silver Stain KANTO III (Kanto Chemical Co.) for silver staining.

### *In vitro* ubiquitination assays

*In vitro* Cul5/Rbx2 (3 µM) neddylation was performed by preincubating APPBP1/UBA3 (0.15 µM) (Boston Biochem), UBE2F (3 µM) (Boston Biochem), Neural precursor cell Expressed Developmentally Down-regulated 8 (NEDD8) (30 µM) (Boston Biochem), ATP (3 mM) (Merck), and the purified Vif/HIS-CBF-β/EloB/C protein complex (3 µM) in Ub buffer (50 mM Tris HCl [pH 7.4], 5 mM MgCl_2_, 1 mM dithiothreitol [DTT], and 1 µM ZnSO_4_) for 60 min at 26°C. Subsequently, UBE2R1 (1.5 µM) (Boston Biochem) and each purified A3 protein, A3G (0.375 µM), or A3-MH protein (0.75 µM), in Ub buffer were added to 5 µL of the Vif/HIS-CBF-β/EloB/C reaction mixture containing neddylated Cul5/Rbx2 to a total volume of 10 µL, followed by incubation for 30 min at 26°C. *In vitro* ubiquitination was performed by the addition of 5 µL of ubiquitin mixture (30 µM ubiquitin [Boston Biochem], 0.2 µM UBE1, 2 mM ATP, 50 mM Tris HCl (pH 7.4), 5 mM MgCl_2_, 1 mM DTT, and 1 µM ZnSO_4_) and incubation for 2 h at 26°C. The ubiquitination reactions were terminated by the addition of 15 µL of 2× Laemmli buffer containing 2.5% 2-ME. The ubiquitinated protein products were separated by SDS–PAGE on a 10% Mini-PROTEAN TGX gel (Bio-Rad) and assessed by immunoblotting with the anti-Myc-tag (9B11) mAb (1:10^3^ dilution; Cell Signaling Technology) for A3C and cA3H or anti-A3G rabbit serum (C-17) (1:10^3^ dilution) (49).

### Structural modeling

We performed 10-ns MD simulations of Vif in complex with CBF-β under explicit water conditions. The initial structure was constructed on the basis of the crystal structure of the VCBC-Cul5 pentameric complex (PDB 4N9F) (3). Because there is no structural information on Vif C-terminal residues 173 through 192, we used the crystal structure of Vif(1-172) as the initial structure. The truncation mutants Vif(1-160) and Vif(1-164) were constructed from this structure by removing C-terminal 8 and 12 residues, respectively. We first performed energy minimization of the CBF-ß/Vif system and subsequently heated the system to 310 K (~37°C). The MD simulations were performed at 310 K at 1 atm using AMBER9 software package (50). The ff10 force field was used to calculate the energies and forces in the simulations. Then, the structural characteristics of 500 trajectories of each model were analyzed with the ptraj module in AMBERTools (http://ambermd.org) from the well-equilibrated 5–10 ns simulations. All the figures containing the obtained molecular structures were drawn with PyMOL (Schrödinger).

## SUPPLEMENTAL MATERIAL

Fig. S1 mbio.03332-24-s0001.pdf Effects of HIV-1 Vif C-terminal truncation on A3F, A3G, and cA3H degradation.

Table S1 mbio.03332-24-s0002.pdf Oligonucleotides used in this study.

ASM does not own the copyrights to Supplemental Material that may be linked to, or accessed through, an article. The authors have granted ASM a non-exclusive, world-wide license to publish the Supplemental Material files. Please contact the corresponding author directly for reuse.
