# Supplementary material for: The unique structure of the highly conserved PPLP region in HIV-1 Vif is critical for the formation of APOBEC3 recognition interfaces
Source: mBio. 2025 Jan 21;16(3):e03332-24. doi: 10.1128/mbio.03332-24 (PMC11898743; doi:10.1128/mbio.03332-24)
Supplement: Fig. S1 — Effects of HIV-1 Vif C-terminal truncation on A3F, A3G, and cA3H degradation. [file mbio.03332-24-s0001.pdf]

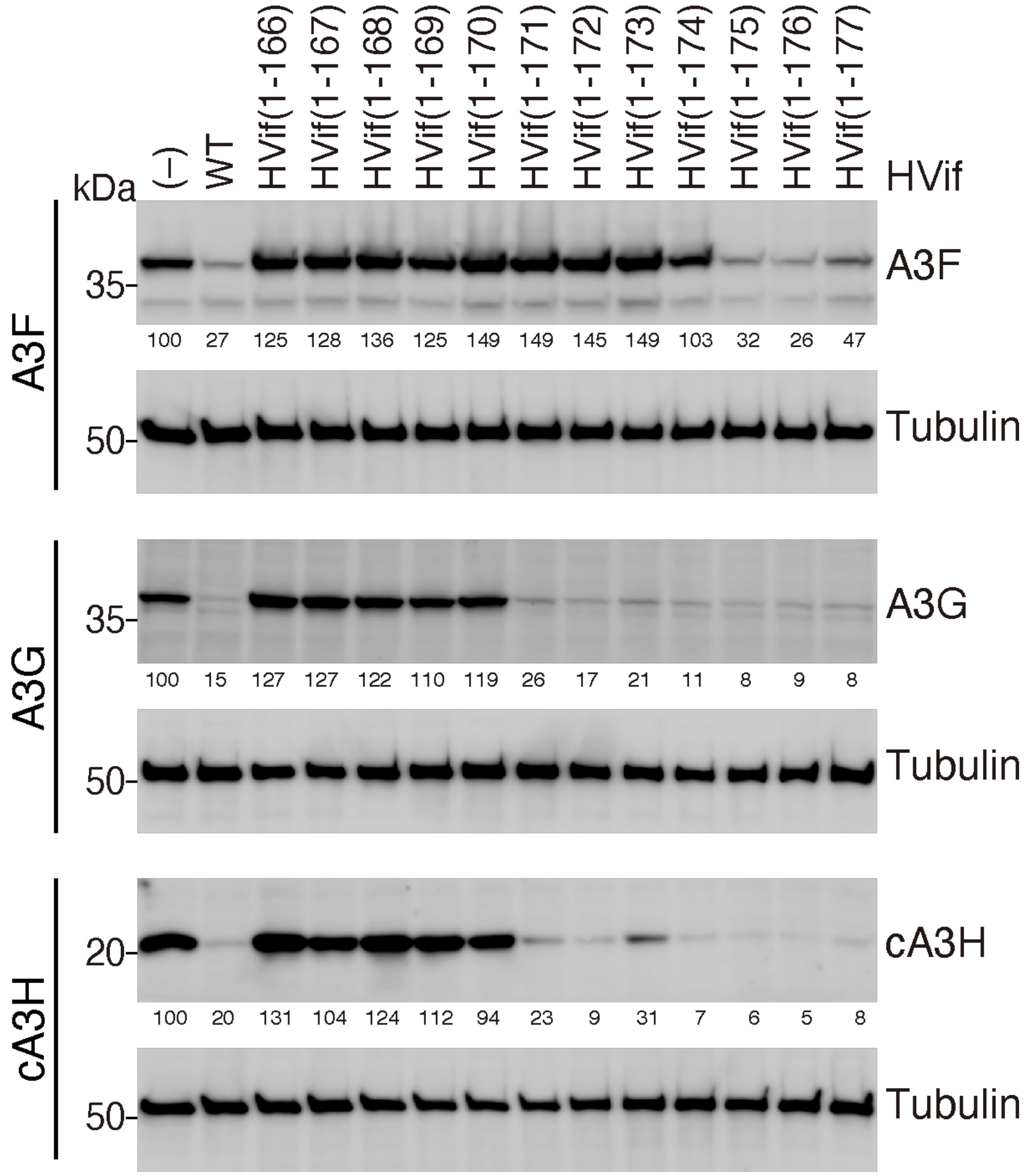

FIG S1 Effects of HIV-1 Vif C-terminal truncation on A3F, A3G and cA3H degradation. A series of C-terminally truncated HVif mutants (without a C-terminal tag) were coexpressed with A3F (+MH tag), A3G (no tag) or cA3H (+FLAG tag) in 293T cells. The intracellular protein levels of A3s were analyzed by immunoblotting with the following antibodies: anti-HIS-tag mAb for A3F; anti-C-17 rabbit serum for A3G; anti-FLAG mAb for cA3H; and anti-ß tubulin rabbit serum. (–) and WT* indicate no Vif and HVif (no tag), respectively. Because there are no available antibodies that can detect truncated HVif mutants, the Vif protein levels were not analyzed by immunoblotting. The percentage (%) of A3 in the presence of HVif relative to that in the absence of HVif was calculated from the immunoblot data and is shown under each A3 image.
