# Supplementary material for: The unique structure of the highly conserved PPLP region in HIV-1 Vif is critical for the formation of APOBEC3 recognition interfaces
Source: mBio. 2025 Jan 21;16(3):e03332-24. doi: 10.1128/mbio.03332-24 (PMC11898743; doi:10.1128/mbio.03332-24)
Supplement: Table S1 — Oligonucleotides used in this study. [file mbio.03332-24-s0002.pdf]

Supplementary Table. S1 Oligonucleotides used in this study

Plasmid Name	Oligonucleotide Name	Oligonucleotide DNA sequences (5´ to 3´)
pUC118 CBF-ß	1st forward: CBF beta (1st) (+)	AAAGGGAAGCGGGCGTCCGGGC
	1st reverse: CBF beta (1st) (-)	AAATAGACCGTAGTACCTCCAG
	2nd forward: CBF beta ATG (2nd) (+)	ATGCCGCGCGTCGTGCCCGACC
	2nd reverse: CBF beta STOP (2nd) (-)	TTAACGAAGTTTGAGGTCATCAC
pcDNA FLAG-CBF-ß	FLAG-CBFb NotI (+)	CTCGAGCGGCCGCATGGATTACAAGGATGACGACGATAAGATGCCGCGCGTCGTGCCCGAC
	M13 Reverse	CAGGAAACAGCTATGAC
pcDNA HVif(1-166)	T7 Promoter	TAATACGACTCACTATAGGG
	HVif (1-166)(+MycHISTag) (-)	AGCTCGGATCCCCCAACACTAGGCAAAGGTGGCTTTAT
pcDNA HVif(1-167)	T7 Promoter	shown above
	HVif (1-167)(+MycHISTag) (-)	AGCTCGGATCCCCCCCTAACACTAGGCAAAGGTGG
pcDNA HVif(1-168)	T7 Promoter	shown above
	HVif (1-168)(+MycHISTag) (-)	AGCTCGGATCCCCCTTTCCTAACACTAGGCAAAGG
pcDNA HVif(1-169)	T7 Promoter	shown above
	HVif (1-169)(+MycHISTag) (-)	AGCTCGGATCCCCCCAGTTTCCTAACACTAGGCAA
pcDNA HVif(1-170)	T7 Promoter	shown above
	HVif (1-170)(+MycHISTag) (-)	AGCTCGGATCCCCCTGTCAGTTTCCTAACACTAGG
pcDNA HVif(1-171)	T7 Promoter	shown above
	HVif (1-171)(+MycHISTag) (-)	AGCTCGGATCCCCCCTCTGTCAGTTTCCTAACACT
pcDNA HVif(1-172)	T7 Promoter	shown above
	HVif (1-172)(+MycHISTag) (-)	AGCTCGGATCCCCCGTCCTCTGTCAGTTTCCTAAC
pcDNA HVif(1-173)	T7 Promoter	shown above
	HVif (1-173)(+MycHISTag) (-)	AGCTCGGATCCCCCTCTGTCCTCTGTCAGTTTCCT
pcDNA HVif(1-174)	T7 Promoter	shown above
	HVif (1-174)(+MycHISTag) (-)	AGCTCGGATCCCCCCCATCTGTCCTCTGTCAGTTT
pcDNA HVif(1-175)	T7 Promoter	shown above
	HVif (1-175)(+MycHISTag) (-)	AGCTCGGATCCCCCGTTCCATCTGTCCTCTGTCAG
pcDNA HVif(1-176)	T7 Promoter	shown above
	HVif (1-176)(+MycHISTag) (-)	AGCTCGGATCCCCCCTTGTTCCATCTGTCCTCTGT
pcDNA HVif(1-177)	T7 Promoter	shown above
	HVif (1-177)(+MycHISTag) (-)	AGCTCGGATCCCCCGGGCTTGTTCCATCTGTCCTC
pNL *vpr*_XhoI	HVif PflMI (+)	GGCGTCTCCATAGAATGGAGG
	NL Vif stop XhoI&EcoRI(-)	TTGCAGAATTCTTATTATGGCTTCCACTCCTGCCCAAGTATCCCCGTAAGTTTCATAGATATGTTGTCCTAAGTTATGGAGCCATATCCTAGGAAAATGTCTAACAGCTTCACTCTTAAGTTCCTCGAGAAGCTCTAGTGTCCATT
pNL Vif (1-169)	1st forward: T7 Promoter	shown above
	1st reverse: HVif (1-169)_NL43(-)	CTCGGATCCGTGTCCATTCATTGTATGGCTCCCTCTGTGGCCCTTGGTCTTCTGGGGCTTGTTCCATCTGTCCTCTCACAGTTTCCTAACACTAGG
	2nd forward: HVif PflMI (+)	shown above
	2nd reverse: NL Vif stop XhoI(-)	AGTTCCTCGAGAAGCTCTAGTGTCCATTCATTGT
pNL Vif (1-170)	1st forward: T7 Promoter	shown above
	1st reverse: HVif (1-170)_NL43(-)	CTCGGATCCGTGTCCATTCATTGTATGGCTCCCTCTGTGGCCCTTGGTCTTCTGGGGCTTGTTCCATCTGTCCTATGTCAGTTTCCTAACACT
	2nd forward: HVif PflMI (+)	shown above
	2nd reverse: NL Vif stop XhoI(-)	shown above
pNL Vif (1-171)	1st forward: T7 Promoter	shown above
	1st reverse: HVif (1-171)_NL43(-)	CTCGGATCCGTGTCCATTCATTGTATGGCTCCCTCTGTGGCCCTTGGTCTTCTGGGGCTTGTTCCATCTCTACTCTGTCAGTTTCCTAAC
	2nd forward: HVif PflMI (+)	shown above
	2nd reverse: NL Vif stop XhoI(-)	shown above
pNL Vif (1-172)	1st forward: T7 Promoter	shown above
	1st reverse: HVif (1-172)_NL43(-)	CTCGGATCCGTGTCCATTCATTGTATGGCTCCCTCTGTGGCCCTTGGTCTTCTGGGGCTTGTTCCATCTTCAGTCCTCTGTCAGTTTCCT

	2nd forward: HVif PflMI (+)	shown above
	2nd reverse: NL Vif stop XhoI(-)	shown above
pNL Vif (1-173)	1st forward: T7 Promoter	shown above
	1st reverse: HVif (1-173)_NL43(-)	CTCGGATCCGTGTCCATTCATTGTATGGCTCCCTCTGTGGCCCTTG GTCTTCTGGGGCTTGTTCCATCACCTGTCCTCTGTCAGTTTC
	2nd forward: HVif PflMI (+)	shown above
	2nd reverse: NL Vif stop XhoI(-)	shown above
pNL Vif (1-174)	1st forward: T7 Promoter	shown above
	1st reverse: HVif (1-174)_NL43(-)	CTCGGATCCGTGTCCATTCATTGTATGGCTCCCTCTGTGGCCCTTG GTCTTCTGGGGCTTGTTCCATCTCTACCACCTGTCCTCTGTCAGTT TC
	2nd forward: HVif PflMI (+)	shown above
	2nd reverse: NL Vif stop XhoI(-)	shown above
pNL Vif L163D	HVif PflMI (+)	shown above
	NL Vif stop XhoI(-)	shown above
pNL Vif V166D	HVif PflMI (+)	shown above
	NL Vif stop XhoI(-)	shown above
pNL Vif L169D	HVif PflMI (+)	shown above
	NL Vif stop XhoI(-)	shown above
pETduet HIS-CBF-ß/HVif	HIS-CBF-ß: TEV-BamH1-CBF-beta-Nter(F)	AGCCAGGATCCGGAGAACCTGTACTTCCAGGGCGCCGGCTCTATG CCGCGCGTCGTGCCCGAC CAG
	HIS-CBF-ß: CBF-beta-Cter-Stop-Hind3(R)	GCCGCAAGCTTCTATTAGGGTCTTGTTGTCTTCTTGCCAGTT
	HVif: HVif ATG(NdeI)(+)	ATATACATATGGAGAACCGGTGGCAGG
	HVif: HVif Stop (XhoI)(-)	CAGACTCGAGCTATTAGTGTCCATTCATTGT
pETduet HIS-CBF-ß/HVif(1-169)	HVif: HVif ATG(NdeI)(+)	shown above
	HVif: HVif (1-169)(noTag) (XhoI) (-)	GAACCTCGAGTTATCACAGTTTCCTAACACTAGGCAA
pETduet HIS-CBF-ß/HVif(1-170)	HVif: HVif ATG(NdeI)(+)	shown above
	HVif: HVif (1-170)(noTag) (XhoI) (-)	GAACCTCGAGTTATCATGTCAGTTTCCTAACACTAGG
pETduet HIS-CBF-ß/HVif(1-171)	HVif: HVif ATG(NdeI)(+)	shown above
	HVif: HVif (1-171)(noTag) (XhoI) (-)	GAACCTCGAGTTATCACTCTGTCAGTTTCCTAACACT
pETduet HIS-CBF-ß/HVif(1-174)	HVif: HVif ATG(NdeI)(+)	shown above
	HVif: HVif (1-174)(noTag) (XhoI) (-)	GAACCTCGAGTTATCACCATCTGTCCTCTGTCAGTTT
pcDNA Cullin5	Cul5 Start (+)	GACTCGAGCGGCCGCATGGCGACGTCTAATCTGTTAAAGAAT
	Cul5 stop (-)	ATCTGTTCTAGATTATGCCATATATATGAAAGTGTTGAT
pCR4 EloB (isoform 1)	EloB start(+)	GACTCGAGCGGCCGCATGGACGTGTTCCTCATGATCCGG
	EloB Stop (-) (for B2)	CACTAAGCTTACAGCCCCCAGCGTGGGTGGAC
pUC118 Rbx2 (isoform 1)	5-Rbx2	AAGCCAACGTCTCCGCCGTCGGCTC
	3-Rbx2	GCTCTGAACAACTGCGCTAAGAAGC
pRSFDuet EloB/C(CO)	EloB: Nco1-EloB-Nter(F)	GATATACCATGGACGTGTTCCTCATGATCCGGC
	EloB: EloB-Cter-Stop-Hind3(R)	GAGCTCAAGCTTCTATTACTGCACGGCTTGTTCATTGGCACTG
	EloC: EloC(CO)ATG(NdeI)(+)	AAGGAGATATACATATGGATGGCGAAGAGAAAAC
	EloC: EloC(CO)stop(XhoI)(-)	CCAGACTCGAGTTACTAGCAGTCGAGAAAGTTGGCAGCC
pRSFDuet HIS-CUL5/RBX2	CUL5: Fwd-BamHI-Cul5v2	GAGAGGATCCGATGGCGACGTCTAATCTGTTAAAG
	CUL5: Cullin5-Cter-Not1(R)	CATTATGCGGCCGCCTATTATGCCATATATATGAAAGTGTTGATA
	RBX2: Fwd-NdeI-Rbx2	GAGACATATGGCCGACGTGGAAGACGGAGAGGAA
	RBX2: Rbx2-Cter-Bgl2(R)	ACCAGAAGATCTCTATTATTTGCCGATTCTTTGGACCACCCAG
pET-21 Rbx2	Fwd-NdeI-Rbx2	GAGACATATGGCCGACGTGGAAGACGGAGAGGAA
	Rbx2-Cter-Not1(R)	ACCAGAGCGGCCGCCTATTATTTGCCGATTCTTTGGACCACCCAG
pEU-E01 A3C-MycHIS	Fwd-EcoRV-A3C	GAGAGATATCATGAATCCACAGATCAGAAACCCG
	Rev-XhoI-myc-A3C	GAGACTCGAGCAGATCCTCTTCTGAGATGAGTTTTTGTTCGGGCC CAAGCTTCTGGAGACTCTCCCGTAGCCTTC
pEU-E01 cpzA3H-MycHIS	Fwd-EcoRV-cpzA3H	GAGAGATATCATGGCTCTGTTAACAGCCGAAAC
	Rev-XhoI-myc-cpzA3H	GAGACTCGAGCAGATCCTCTTCTGAGATGAGTTTTTGTTCGGGCC CAAGCTTGGACTGCTTTATCCTCTCAAGCC
